# Video-assisted transcervical-transtracheal repair of posterior wall laceration of thoracic trachea: A new approach. Case Report

**DOI:** 10.3389/fsurg.2023.1120404

**Published:** 2023-02-08

**Authors:** Simone Tombelli, Domenico Viggiano, Lavinia Gatteschi, Luca Voltolini, Alessandro Gonfiotti

**Affiliations:** Division of Thoracic Surgery, Careggi University Hospital, Florence, Italy

**Keywords:** tracheal laceration, cervicotomy, hybrid approach, case report, thoracoscopy (VATS)

## Abstract

Iatrogenic tracheal lacerations are a rare but potentially fatal event. In selected acute cases, surgery plays a key role. Treatment can be conservative, for lacerations of less than 3 cm; surgical or endoscopic, depending on the size and location of the lesion and fan efficiency. There is no clear indication of the use of any of these approaches and the decision is therefore linked to local expertise. We present an emblematic clinical case of a 79 years old female patient undergoing polytrauma as a result of a road accident, without neurological damage, which required intubation and subsequent tracheotomy due to a significant limitation to ventilation. Imaging has shown the tracheal laceration involving the anterior wall and the pars membranacea up to the origin of the right main bronchus.A percutaneous tracheotomy was permormed without any improvement of the respiratory dynamic. Therefore, the patient underwent a surgical repair of the tracheal laceration with a hybrid mini-cervicotomic/endoscopic approach. This less invasive approach successfully repaired the extensive loss of substance.

## Introduction

The trachea is an unequal median organ that, following the larynx, ends in the thorax bifurcating in the main right and left bronchus. In its course it makes contact with multiple structures such as the esophagus, thyroid, major arterial and venous vessels of the mediastinal region and neck, nerve structures such as the recurrent laryngeal nerves and the vagus nerve and, posteriorly, with the vertebra and spinal cord (1).

It is therefore clear why the majority of patients who suffer tracheal injuries can exhale before arrival in emergency facilities, given the proximity of all these vital structures. The true incidence of trachea bronchial injuries (TBI) is still unknown since 30%–80% of these trauma victims still die at the scene of the accident (2). Currently, the incidence of TBI among trauma patients with chest and neck injuries is between 0.5% and 2%. Major causes of tracheal injury include trauma (contusive and penetrating) and iatrogenic injury. Considering endotracheal intubations, which represent one of the leading iatrogenic causes, iatrogenic trachea lesions occur once every 20,000–75,000 elective intubations and increase up to 15% for intubations performed in emergency (2). Tracheal lacerations differ depending on the involvement of the cervical region or thoracic region, the impairment of the anterior wall or the pars membranacea, and if the tracheal wall is involved partially or at full thickness.

For all the reasons listed above, early detection is one of the most important factors to reduce morbidity and mortality related to this injury. Proper airway management is vital (3). Despite the high mortality and morbidity of this kind of injuries there is still not a unanimous expert consensus on the most proper treatment to apply and every center relies on its expertise. In this article we would like to depict an extremely severe case of tracheal laceration and our innovative method to repair it.

## Case description

We present a clinical case of a 79-year-old patient subjected to polytrauma following a car accident, without obvious neurological damage, which required intubation at the scene of the accident performed by the emergency service personnel.

The patient was taken to the emergency room of the Careggi University Hospital and was then admitted to the intensive care unit (ICU) where, due to an increasing and significant limitation to ventilation the patient was sedated and muscle relaxers were used. Eventually a percutaneous bed-side tracheotomy had to be performed in order to achieve a proper ventilation management with no immediate periprocedural complications. Nevertheless, during the ICU stay there was a progressive difficulty in mechanical ventilation with evidence of air leakage from the ventilation circuit. Chest x-ray did show neither pneumothorax nor subcutaneous emphysema, therefore a direct thoracic computed tomography (CT) scan was performed and it revealed a tracheal tear extending from about 5 cm from the glottis up to the carena, involving the origin of the right main bronchus. A fibrobronchoscopy was taken and confirmed the extent of the laceration and showed the esophagus bulging through the mediastinum into the trachea. Considering the wide loss of substance, and the likely iatrogenic nature of the lesion, a conservative approach was impossible to carry out and the multidisciplinary decision was to perform a surgical procedure. Since there was no pneumothorax we opted for a cervical approach.

**Figure 1:**
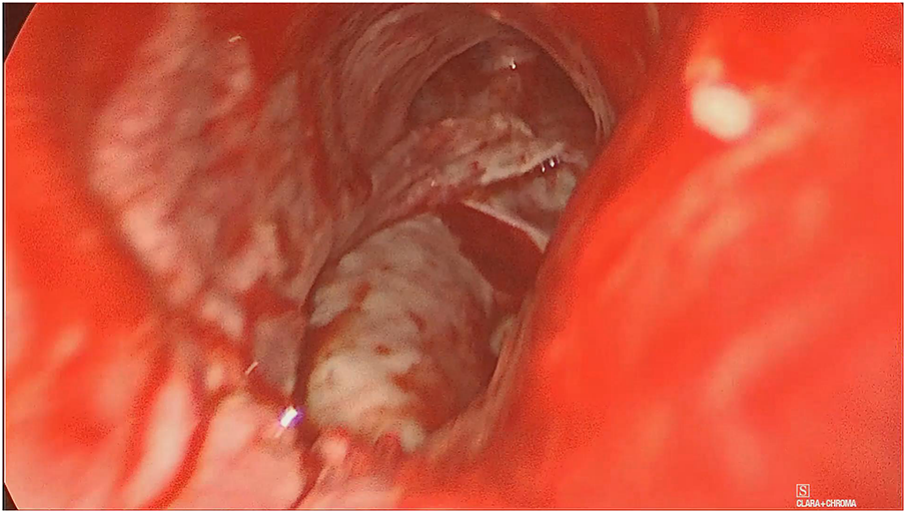
Lesion of the pars membranacea extending from 5 cm from the glottis up to carena, including the origin of the main right bronchus. Bulging of the anterior esophageal wall is visible through the loss of trachea substance.

The patient was taken to the operating room where mini-cervicotomic surgical access comprehending the previous transcutaneous tracheotomy allowed us to reach the thyroid plane with subsequent isolation and section of the isthmus. The anterior wall of the trachea was damaged as well as the posterior one. There were broken down fractures with loss of substance of cricoid cartilage and the first four tracheal rings, determining the opening of the airway from the base of the larynx up to the upper third of the trachea. In order to verify and manage the whole length of the laceration we decided to use a 5 mm 30° degrees thoracoscope inserted through the cervicotomic access. It magnified and showed us the known lesion of the pars membranacea, 5 cm from the glottis up to the carena and involving the origin of the right main bronchus. The tear was confirmed to be at full thickness with bulging of the esophagus and large virtual space between the anterior esophageal wall and trachea, resulting in a false lumen produced by the tracheostomic cannula (Figure 1). The pars membranacea in the posterior right portion was retracted with multiple lacerations and consequent loss of substance.

**Figure 2:**
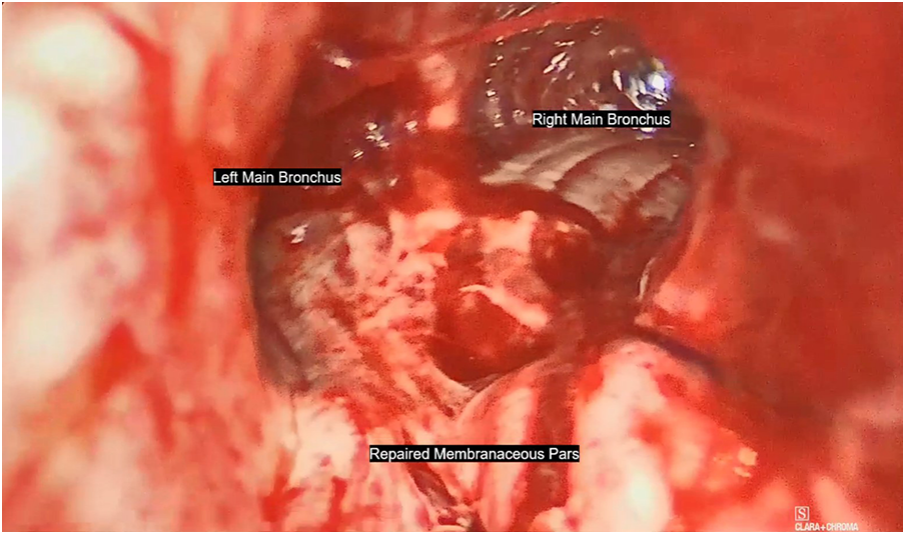
The pars membranacea was repaired with continuous suturing in 3-0 absorbable monofilament Polydiossanone while the anterior tracheal wall was sutured with single 2-0 Polyglactin braided absorbable stitches using cervicotomic access.

Transtracheal repair was carried out with video-assisted technique in intermittent apneic ventilation after partial anterior opening of the remaining pars cartilaginea. The pars membranacea was repaired with continuous suturing in 3-0 absorbable monofilament Polydiossanone while the anterior tracheal wall was sutured with single 2-0 Polyglactin braided absorbable stitches using cervicotomic access (Figure 2). Thanks to the scope we managed to repair the whole posterior wall laceration through the cervicotomy, with no need of entering the chest.

The posterior wall was successfully repaired but there still was an important alteration of the tracheal anatomy. At the end, the loss of substance due to the fractures of tracheal rings and the cricoid forced us to pack a, tracheotomy in order to protect the airway. Proper functioning of the mechanic ventilation and no air leakage were detected. Operation time was 180 min. This surgical approach let a quick visualization of the lesion, amplifying the images. The only use of the cervical access has also allowed us to avoid the sternotomic or thoracotomic access with advantages in terms of trauma and operation time.

## Discussion

When a conservative approach is not feasible, the standard surgical approach should be the cervicotomy in the case it was injured the 2/3 superior trachea and the larynx, as first described by Angelillo-Mackinlay (4), or the thoracotomy if the trauma involves the 1/3 inferior trachea. For tracheal lesions occurred to the carena or involving the origin of the main bronchi the approach in anterolateral thoracotomy or right posterolateral thoracotomy can be used. If the lesion distally affects the left main bronchus, this could be addressed through a left posterior thoracotomy if necessary. When increased exposure of the hemithorax or mediastinum is required, such as in the case of a suspected intrapericardial injury, a median partial sternotomy or clamshell incision could be used, even though this does not provide better access to the trachea (5).

As described by Grillo and colleagues (6) tracheal dissection around the lesion must be performed meticulously and closed along the trachea to minimize the risk for laryngeal nerve injury. Tracheal wall should be sutured with running suture in absorbable monofilament (7).

**Figure 3:**
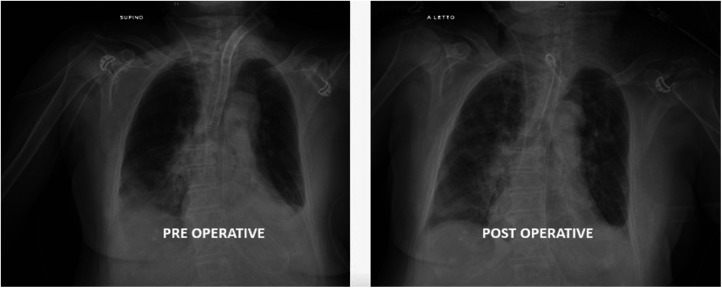
An adequate tracheal caliber and the presence of the new tracheostomic cannula can be appreciated at the post-operative x-ray. The mechanical ventilation system showed no air losses in the circuit at the end of surgery.

Our hybrid surgical approach allowed us to repair the posterior laceration with a running suture in 3-0 absorbable monofilament Polydiossanone under visual control using the endoscopic camera while the anterior tracheal wall was sutured with single 2-0 Polyglactin braided absorbable stitches entirely using the cervicotomic access under direct vision while maintaining an adequate caliber of the airway (Figure 3). In addition, the association of endoscopy with traditional surgery has favored a less invasive approach, compared with the techniques described above, as proposed by Mussi et al. (8). Unfortunately, the patient died four days after the surgery due to unforeseen complications related to organ damage reported as a result of the trauma, unrelated to airway problems.

## Data Availability

The original contributions presented in the study are included in the article/**Supplementary Material**, further inquiries can be directed to the corresponding author/s.
