# Supplementary figures and images for: Video-assisted transcervical-transtracheal repair of posterior wall laceration of thoracic trachea: A new approach. Case Report
Source: Front Surg. 2023 Feb 8;10:1120404. doi: 10.3389/fsurg.2023.1120404 (PMC9945533; doi:10.3389/fsurg.2023.1120404)

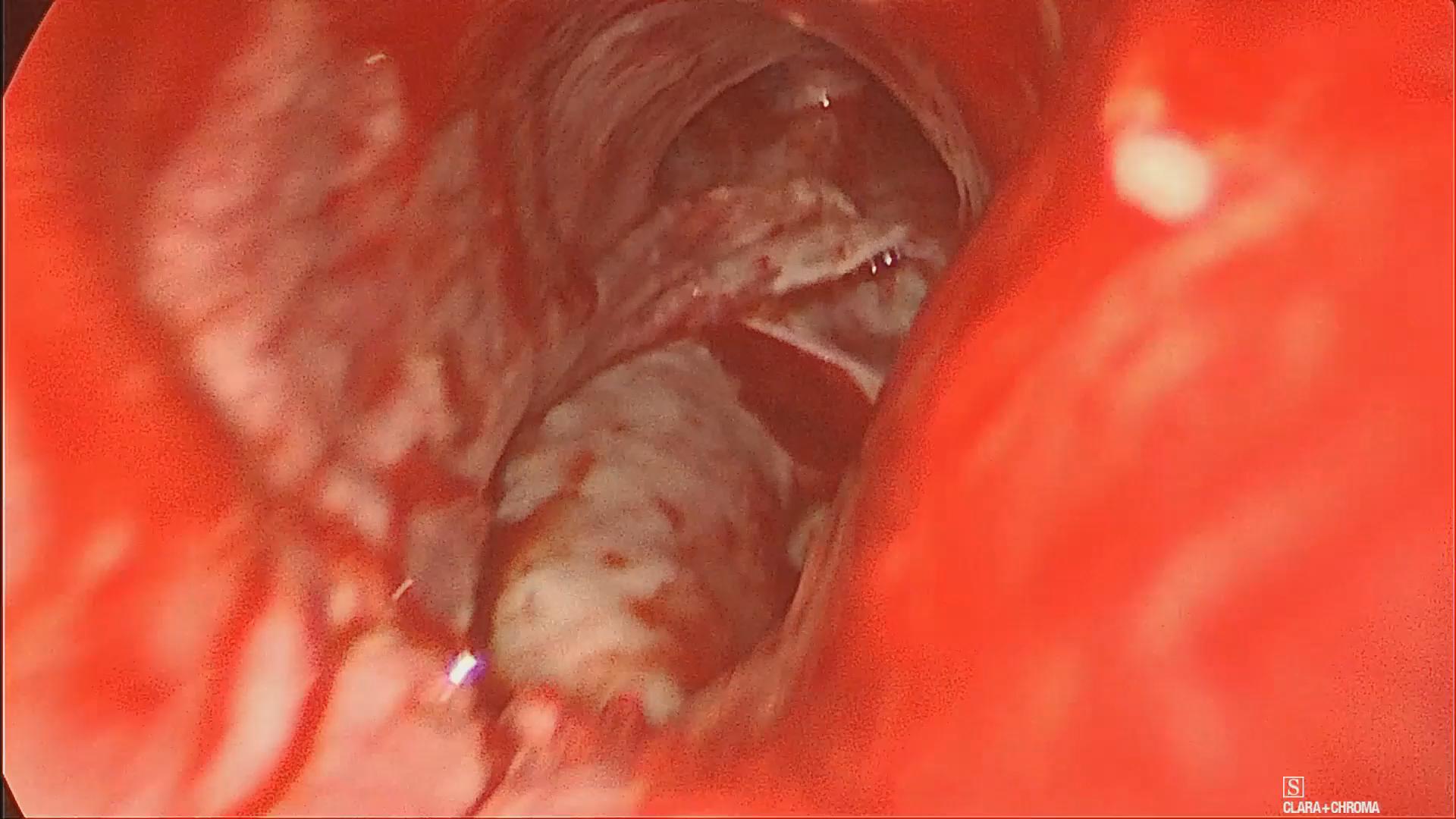

Supplement: Supplementary file 1 [file Datasheet1.zip › Data Sheet 1_v1/supplementary material/Figure 1.JPEG]

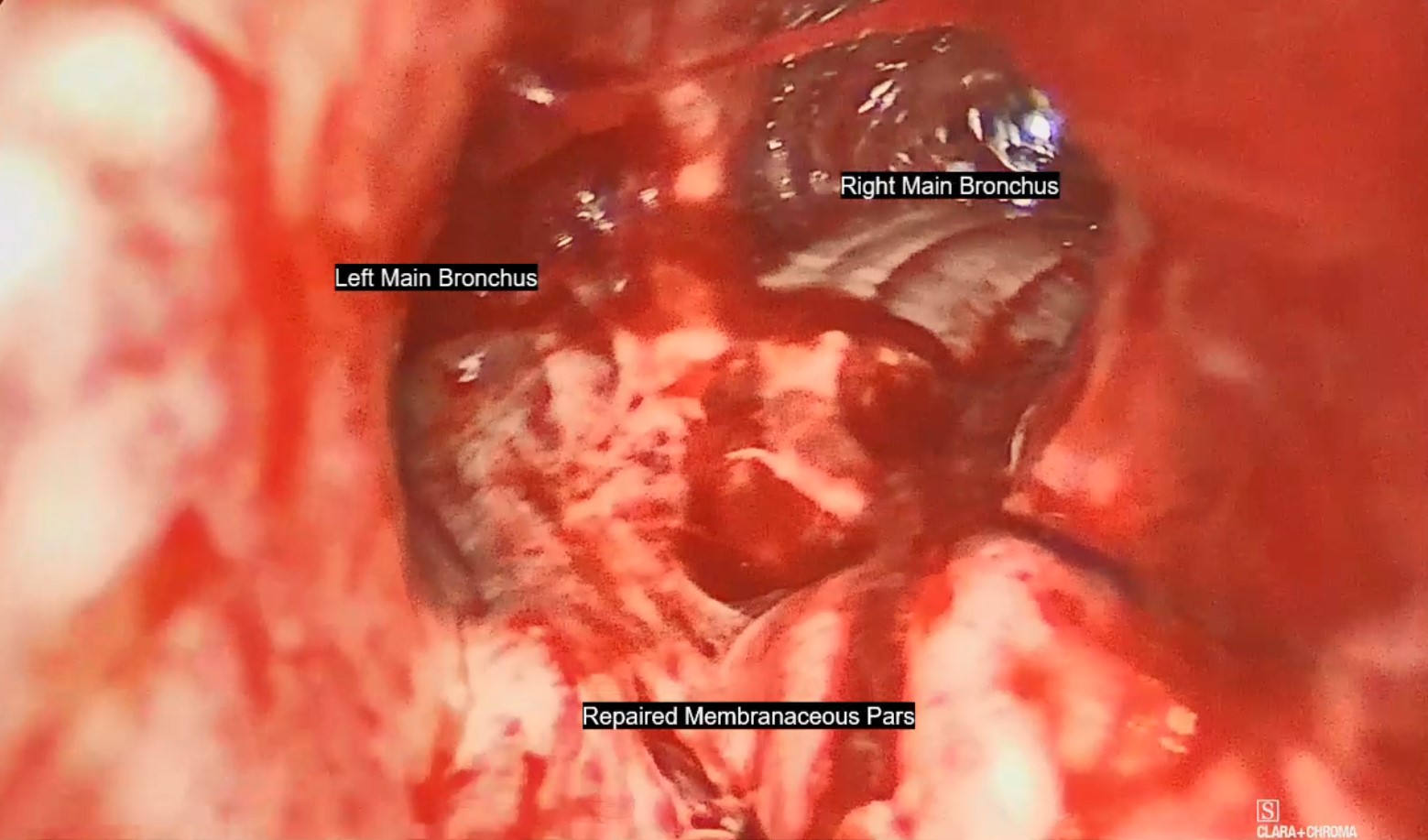

Supplement: Supplementary file 1 [file Datasheet1.zip › Data Sheet 1_v1/supplementary material/Figure 2.JPEG]

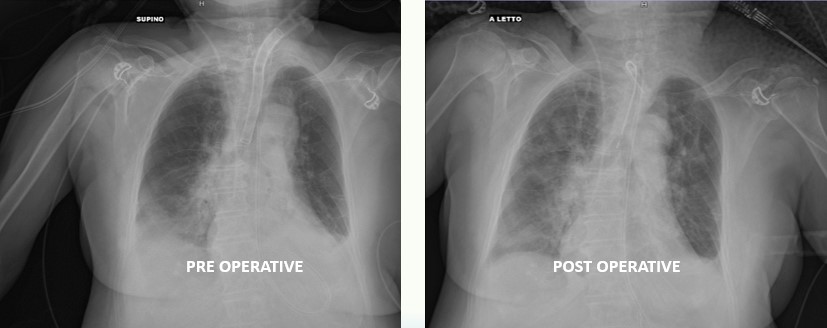

Supplement: Supplementary file 1 [file Datasheet1.zip › Data Sheet 1_v1/supplementary material/Figure 3.JPEG]
